# Comparison of the Antimicrobial Effect of Atorvastatin and Nano-Atorvastatin Mouthwash on *Aggregatibacter Actinomycetemcomitans*: An *in vitro* Study 

**DOI:** 10.30476/dentjods.2025.103864.2483

**Published:** 2026-06-01

**Authors:** Atefeh Ramezani, Tahura Etezadi, Hamidreza Goli, Nafas Daraei, Majid Saeedi, Melika Mollaei, Alireza Sedighi, Abolfazl Hosseinnataj, Hodis Ehsani

**Affiliations:** 1 Dept. of Prosthodontics, Dental Research Center, Faculty of Dentistry, Mazandaran University of Medical Sciences, Sari, Iran.; 2 Dept. of Orthodontics, Dental Research Center, Faculty of Dentistry, Mazandaran University of Medical Sciences, Sari, Iran.; 3 Dept. of Medical Microbiology and Virology, Faculty of Medicine, Mazandaran University of Medical Science, Sari, Iran.; 4 Student Dentistry, Dental Research Center, Student Research Committee, Faculty of Dentistry, Mazandaran University of Medical Sciences, Sari, Iran.; 5 Pharmaceutical Sciences Research Center, Haemoglobinopathy Institute, Mazandaran University of Medical Sciences, Sari, Iran.; 6 Dept. of Biostatistics, Faculty of Health, Mazandaran University of Medical Sciences, Sari, Iran.

**Keywords:** Atorvastatin, Nanoparticle, Periodontitis, *Aggregatibacter Actinomycetemcomitans*

## Abstract

**Background:**

The emergence of resistance, side effects, and the high cost of drugs indicates a need for other therapeutic alternatives with similar properties but fewer side effects.

**Purpose:**

The present study aims to compare the antimicrobial effect of atorvastatin and nano-atorvastatin mouthwash on *Aggregatibacter Actinomycetemcomitans*.

**Materials and Method:**

In this *in vitro* study, the atorvastatin and nano-atorvastatin mouthwashes were prepared, and their antibacterial property against *Aggregatibacter Actinomycetemcomitans* was assessed using the agar well diffusion and microbroth dilution tests. Water and chlorhexidine were considered as the negative and positive control groups. Kruskal-Wallis and Mann-Whitney tests were used to compare the size of the diameter of the non-growth halo. Data analysis was obtained using SPSS V.22, and the significance level was considered less than 0.05.

**Results:**

The results of the well diffusion test showed that the diameter of the non-growth halo of chlorhexidine, atorvastatin, and nano-atorvastatin was 27, 18, and 12mm, respectively. The diameter of the halo of non-growth among different substances was statistically significant (*p* Value=0.08), however, no significant difference was observed between atorvastatin and nano-atorvastatin (*p*= 0.05). The findings of the microbroth dilution test showed that atorvastatin and nano-atorvastatin had the minimum inhibitory concentration of 0.039 and 0.002 µg/ml, respectively.

**Conclusion:**

The present study suggests strong antimicrobial activity of atorvastatin and nano-atorvastatin against *Aggregatibacter Actinomycetemcomitans*. Therefore, these substances can be used as an additional drug in treating periodontal diseases.

## Introduction

Periodontal disease is a chronic inflammatory condition that is one of the main oral health issues affecting adults in both developed and developing countries [ 1 ]. Emerging evidence suggests that the inflammatory response induces changes in the periodontal microbiome and promotes the pathogenesis of progressive periodontitis. It is commonly known that bacteria, particularly gram-negative anaerobic bacteria like *Aggregatibacter Actinomycetemcomitans* (Aa), are closely associated with periodontal disease [ 2 ].

Aa is one of the most important periodontal pathogens present in microbial plaque, which can induce host inflammatory mediators that lead to collagen destruction of connective tissue and alveolar bone loss. If left untreated, the progression of infection foci leads to the destruction of the periodontium, followed by tooth mobility and loss, and related cosmetic and functional consequences, which impose a high cost on patients [ 3 ].

Periodontal diseases are treated with a combination of surgical procedures, antimicrobial medications, root planing, scaling, and subgingival scaling, and daily oral hygiene [ 4 ]. Mechanical plaque control by brushing and flossing is the most recommended method for maintaining oral and periodontal health. However, this technique is inconvenient for the patients and fails to reduce bacteria located in the dentinal tubules and grooves [ 5 ].

In addition to mechanical plaque control, different antimicrobial agents are incorporated into chemical plaque control products, such as mouthwashes and toothpastes. Chemical plaque control agents have a good content in the oral cavity, which enables them to maintain oral hygiene between brushings. It is noteworthy that some of these products are susceptible to constraints, such as the development of resistant bacteria that limit their utilization [ 6 - 7 ]. Currently, the most effective disinfectant for the chemical management of microbial plaque is chlorhexidine (CHX), but prolonged use can have negative consequences as well, including discoloration of teeth and restorations, changes in the sense of taste, dry mouth, and allergic reactions [ 8 ]. 

The use of statins reduces the possibility of developing periodontal disease. Statins have recently been introduced as a potential drug therapy. Statins are inhibitors of 3-hydroxy-3-methylglutaryl COA reductase molecules, thus reducing the production of very-low-density lipoprotein (VLDL) and increasing the elimination of low-density lipoprotein (LDL). They are also used to reduce cholesterol and fat. These compounds affect bone metabolism through the production of isoprenoids and C-reactive proteins [ 9 - 10 ]. These agents are suggested to have antioxidative, anti-inflammatory, antibacterial, and immunomodulatory properties [ 11 ]. Moreover, new investigations have reported that statins are beneficial for periodontal health [ 12 - 13 ].

Nano dentistry is the term for the application of nan-otechnology to dentistry. This emerging field is revolutionizing dentistry by introducing novel nanomaterials for enhanced diagnostics, more effective treatments, and improved products for maintaining oral health. Advanced discoveries in the field of nano dentistry are being made, including the utilization of metals, minerals, natural polymers, and medications in general. These resources contribute to the ongoing study of nano dental additives in orthodontics, regeneration, and prostheses [ 14 ].

Clinical research on patients with chronic periodontitis has recently recommended the use of simvastatin or atorvastatin as a supplemental treatment; such progress has been greater in the case of atorvastatin. One of the conducted studies showed that the minimum inhibitory concentration (MIC) of atorvastatin against Aa was 12.5 μg/ml [ 15 - 16 ]. However, high doses of statins cause side effects such as myotoxicity with myopathy and destruction of striated muscle cells [ 17 ]. Considering the gap in the available information on the effect of using mouthwash containing atorvastatin on Aa, as well as the shortcomings of the current common methods, this study aims to compare the effect of atorvastatin mouthwash and nano-atorvastatin on Aa. 

## Materials and Method

This *in vitro* study was conducted in the Microbiology Laboratory of Mazandaran University of Medical Sciences (IR.MAZUMS.REC.1402.18083).

Aa strains were obtained from the microbiology department of the Mazandaran Faculty of Medicine. The bacteria were cultivated in brain heart infusion agar medium (Merck, Germany) and incubated at 37°C in an anaerobic jar (Whitley Jar Gassing System- Don Whitley Scientific, Germany) in an oxygen-free environment produced by the Anoxomat device [ 18 ].

Aa was transferred to thioglycolate broth medium using a paper point. The samples were immediately analyzed in the bacteriology laboratory of Mazandaran University of Medical Sciences. The samples were cultured in the brain heart infusion agar containing 9mg/ml vancomycin, 5gr/l yeast extract, 1mg/ml vitamin K1, 5% sheep blood, 1.5g/l sodium fumarate, and 1g/l sodium formate. The culture medium was incubated at 37°C for 48 hours under anaerobic conditions. Star-shaped colonies indicating gram-negative bacilli were evaluated. A resistance test to kanamycin 500mg was performed for isolates, and their identity was determined [ 19 ].

**Figure 1:**
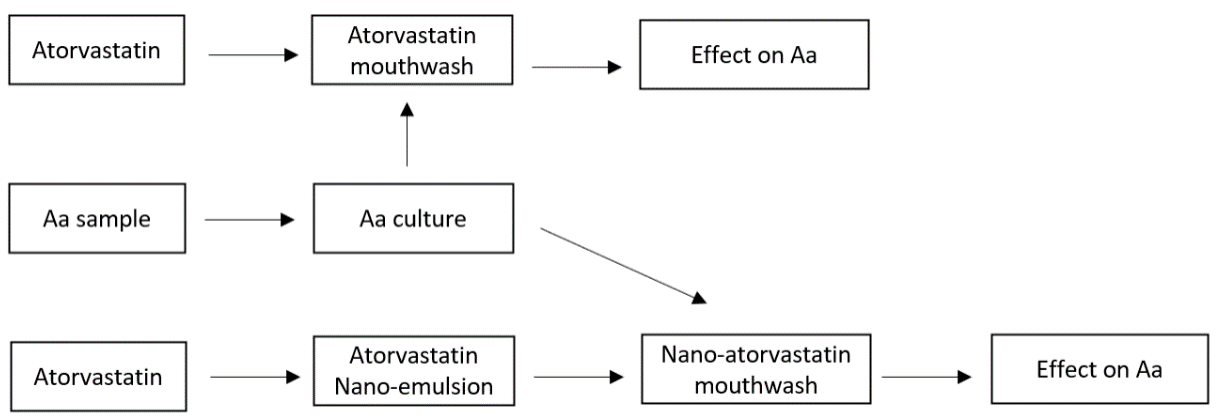
The flow chart of the study

Pure atorvastatin drug powder (Kimiya Acid, Tehran, Iran) was purchased and tested using microbial analysis to be free of any bacteria, yeast, or mold. Atorvastatin mouthwash was made using solvents (water and alcohol), a viscosity-increasing agent (glycerin), a sweetening agent (sodium saccharin), an antimicrobial agent, antimicrobial preservative agents (methylparaben and propylparaben), and an emulsifier (propylene glycol). Figure 1 illustrates the flow of the study. 

A nanoemulsion was prepared using the ultrasonication technique and non-ionic surfactants such as Tween 80 and Span 80 [ 20 ]. At first, the organic phase, including atorvastatin, cholesterol, Tween 80, and Span 80, was placed on the heater stirrer until the temperature reached 70°C so that they were uniformly melted. Then the liquid phase containing water was placed on the heater stirrer and heated to at least 70°C. Once the phases were homogenized and reached thermal equilibrium, the aqueous phase was added to the organic phase. The phases were then mixed for 10 minutes under rotation at 2000 rpm. Finally, to reduce the size and dispersion of the obtained emulsion particles (making nanoparticles), a homogenizer probe was used with a power of 20% for 3 minutes [ 21 ].

### Determination of particle size, dispersion index, and zeta potential

The Zetasizer Nano ZS system (Malvern Instruments, Worcestershire, UK) was used to determine the particle size, polydispersity index (PDI), and zeta potential of the obtained particles using the dynamic light scattering (DLS) technique with a 90° angle at the surface of the particles at room temperature (25°C) was used. For each formulation, three separate samples were obtained, and each sample was repeated three times at room temperature (25°C) without dilution [ 22 ].

### Entrapment Efficiency (EE)

The centrifugation method was used to evaluate the amount of atorvastatin encapsulated in niosomal vesicles. Centrifugation of niosomal dispersions was performed at 18000 rpm for 30 minutes at 4°C (SIGMA 3- 30KS refrigerated centrifuge, Germany). The superficial layer was filtered (pore size 0.22μm), and then atorvastatin in the filtered solution (free drug) was detected using a UV spectrophotometer at 238nm. All experiments were performed at 25°C. The EE of a drug can be calculated with the following equation: 

EE%= [(weight of initial drug−weight of free drug)/ weight of initial drug] *100

### Scanning Electron Microscopy (SEM)

Scanning electron microscopy (SEM, FE-SEM TESCA-NMIRA3, Czech) was used to evaluate the morphology of optimal niosomes (Atrosomes-2). A drop of the sample was placed on a carbon-coated copper grid, and then the sample was air-dried and coated with gold to make the sample conductive. The images were accelerated with a voltage of 20 kV [ 23 ]. 

ATR-loaded niosomes were successfully fabricated using ultrasound. Particle size exhibited a significant inverse relationship with cholesterol content (spanning 144.7±13.6nm at a 1:10 cholesterol: surfactant ratio to 351.9±22.4nm at a 1:2 ratio; *p*< 0.001). EE was consistently high across formulations (80.96±1.65% to 87.18± 0.14%; *p*< 0.001), modulated by cholesterol's effect on membrane permeability. The PDI ranged from 0.45± 0.06 to 0.98±0.03 (*p*< 0.001), with the lowest PDI observed at the lowest cholesterol concentration. Inclusion of cholesterol caused a slight, non-significant reduction in zeta potential (*p*> 0.05), though formulations demonstrated good stability. 

### Atorvastatin nanoemulsion mouthwash preparation 

Nano-atorvastatin was a lyophilized powder. For the preparation of nano-atorvastatin mouthwash, water was used as a solvent due to the structural nature of niosome nanoparticles. Other components of the formulation were methyl paraben, propylparaben and sodium saccharin.

**Figure 2:**
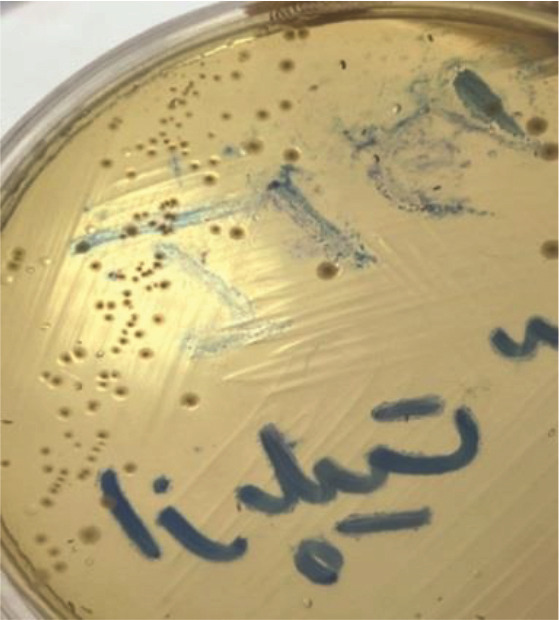
Bacteria cultured in brain heart infusion agar medium

In this study, well-diffusion and micro broth dilution methods were used to investigate the effect of mouthwash on Aa. To perform the well-diffusion test, a suspension equivalent to half McFarland's turbidity in normal saline was prepared from bacteria grown in anaerobic conditions. This suspension contains 1.5×108CFU/ml of bacteria and has an absorbance equal to 0.08 to 0.13 at the wavelength of 625nm. The 0.5 McFarland standard prepared using a sterile cotton swab was cultured on the surface of brain heart infusion agar culture medium in the form of grass (Figure 2). The well-diffusion test was repeated three times, and the average was reported. 

In this experiment, wells with a diameter of 6mm were created on the culture medium using a heat-sterilized glass Pasteur pipette. In these created wells, 50 microliters of atorvastatin with a concentration of 5mg/ml, nano-atorvastatin with a concentration of 5mg/ml, carrier, water as a negative control, and 0.2% CHX (Najo, Tehran, Iran) were added as a positive control. The plates were incubated under anaerobic conditions and at a temperature of 37°C, and after 48 hours, the diameter of the non-growth halos around the discs was measured with a ruler. This method was repeated three times [ 18 ].

In the MIC determination method by microbroth dilution test, atorvastatin was prepared with an initial concentration of 30μg/ml, and nano-atorvastatin with an initial concentration of 80μg/ml. By using the broth microdilution method; working solutions were prepared with double dilutions of antibiotics. After 2 times dilution, concentrations of 7.5 micrograms/ml of atorvastatin and 20 micrograms/ml of nano-atorvastatin and CHX mouthwash solution with a concentration of 0.05% were prepared. In this test, which was performed in a 96-well U-shaped microplate, each row was assigned to one inhibitor. First, 100 microliters of brain heart infusion broth (Merck) were added to all the wells. Then, 100 microliters of the corresponding inhibitor were added to the first well of each row, and after mixing by the sampler, 100 microliters of it were transferred to the next well, and this continued until the tenth well. The last 100μl was transferred to the twelfth well as a negative control. The positive control well contained the desired microbial stock and culture medium, and the negative control well contained the initial drug stock and culture medium. In the positive control well, turbidity (bacterial growth) was observed, and the negative control well was without bacterial growth. The MIC test was repeated twice, and the average was reported.

In the next step, half of McFarland's suspension was prepared from bacteria and diluted 1:100, and 100 micr-oliters were added to all wells (except for well number 12). Well number 11 was considered a positive control. Finally, in the 10th well, a concentration of 0.014μg/ml for atorvastatin, 0.039μg/ml for nano-atorvastatin, 0.000 -3% for CHX (in the 8th well), and a concentration of 0.195% for the carrier (used in mouthwash) was obtained.

The microplates were placed on the shaker for 30 seconds to make the mixture completely uniform. The microplates were placed in an incubator at 35°C under anaerobic conditions for 18 hours. The lowest dilution of the drug in which no turbidity was seen was considered the MIC. The turbidity of the wells was compared with the turbidity of the positive control well, and the clarity of the wells was compared with the negative control well. Finally, the obtained results were compared with the existing standards in Clinical and Laboratory Standards Institute (CLSI) [ 24 - 25 ].

Mean, standard deviation, and median indices were used to describe the variables. Kruskal-Wallis and Mann -Whitney tests were used to compare the size of the growth halo diameter in different materials. Data analysis was done through SPSS version 22 software, and the significance level was considered less than 0.05. 

## Results

**Figure 3:**
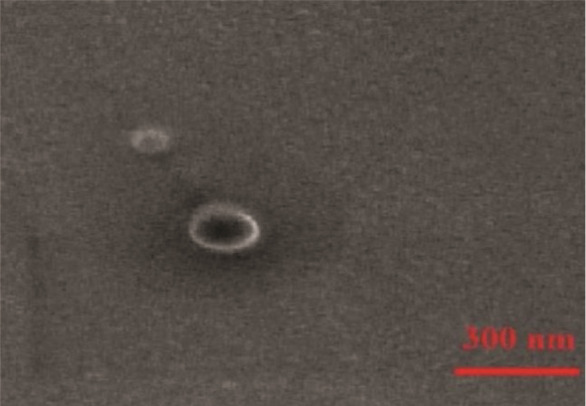
Micrographs of atrosome-2 under scanning electron microscope

Based on optimal characteristics- smaller size (196.33± 6.45 nm), high EE (86.16±0.59%), favorable zeta potential (-20.73±0.98mV), and low PDI (0.46±0.05)- Atros-ome-2 (cholesterol: surfactant ratio 1:5) was selected for further study. SEM analysis confirmed the spherical morphology and absence of aggregation in Atrosome-2 (Figure 3), corroborated by DLS hydrodynamic diameter measurements.

The results of well-diffusion and microbroth dilution tests are described in Table 1, in which the highest and the lowest amounts were for CHX and water and carrier, respectively. Additionally, the findings suggested a statistically significant difference between the substances regarding the diameter of the halo of non-growth bacteria (*p*< 0.05). 

**Table 1:** Comparison of the diameter size of the halo of non-growth of bacteria against the tested antimicrobial substances

Substance	Mean (mm)	SD	Median	Mean rank	Kruskal-Wallis test	*p* Value
atorvastatin	18.00	2.000	18.00	11.00	13.68	0.008
Nano-atorvastatin	12.00	1.000	12.00	8.00		
carrier	0.00	0.000	0.00	3.50		
water	0.00	0.000	0.00	3.50		
CHX	27.00	2.646	26.00	14.00		

SD= Standard Deviation

**Figure 4:**
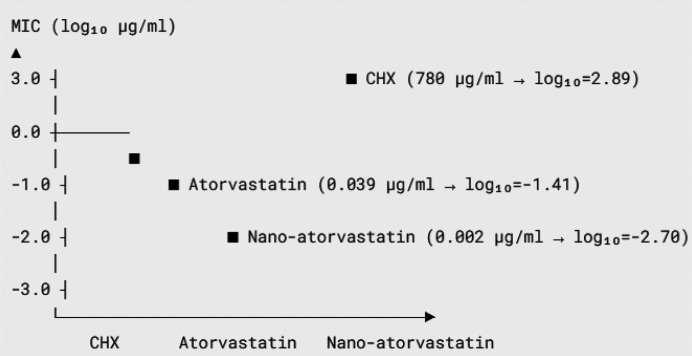
The minimum inhibitory concentration (MIC) of the study substances

Considering the significant difference between different materials, two-by-two comparisons of materials were used to find significant differences (Table 2). The results reported a statistically significant difference between atorvastatin, nano-atorvastatin, and CHX with water and carrier. The rest of the comparisons had no significant difference. Furthermore, the MIC of the substances is reported in Table 3 and Figure 4. 

**Table 2:** Comparison of the diameter of the inhibition zone in the study substances

Variables		Mean rank	Mann-Whitney statistics	*p* Value
Atorvastatin	Nano-atorvastatin	2.00	1.96	0.050
	Carrier	2.00	2.09	0.037
	Water	2.00	2.09	0.037
	CHX	5.00	1.96	0.050
Nano- atorvastatin	Carrier	2.00	2.09	0.037
	Water	2.00	2.09	0.037
	CHX	5.00	1.96	0.050
Carrier	Water	3.50	-	-
	CHX	5.00	2.09	0.037
Water	CHX	5.00	2.09	0.037

CHX= Chlorhexidine

**Table 3:** The minimum inhibitory concentration (MIC) of the study substances

Substance	Well number	MIC
atorvastatin	6	0.039 µg/ml
Nano-atorvastatin	10	0.002 µg/ml
carrier	-	-
water	8	0.003 %
CHX	5	0.078 %

MIC=minimum inhibitory concentration, CHX= Chlorhexidine

## Discussion

The current study investigated the antibacterial effect of atorvastatin and nano-atorvastatin mouthwash on Aa and revealed compelling antimicrobial activity against Aa for both conventional atorvastatin (MIC: 0.039µg/ ml) and its nano-formulated counterpart (MIC: 0.002µg/ ml). The observed 20-fold greater potency of nano-atorvastatin represents a significant advancement over existing formulations. This substantial MIC reduction occurred despite nano-atorvastatin producing a smaller inhibition zone (12mm) than conventional atorvastatin (18mm) in diffusion assays- a finding requiring careful interpretation. Methodological considerations explain this apparent discrepancy; while diffusion tests measure radial compound dispersion through semisolid media, MIC assays directly assess bacterial growth inhibition in liquid environments. The nano-formulation's larger hydrodynamic size and complex surfactant composition demonstrably restrict agar, masking its true potency in diffusion-based assessments [ 26 ]. This phenomenon aligns with observations by Fan *et al*. [ 27 ], who reported that homogenous spherical nano-encapsulated simvastatin particles with a lower size are likely to show better antimicrobial effects.

Statins are HMG-CoA enzyme inhibitors that prevent the formation of an intermediate product (mevalonate). Lack of mevalonate inhibits protein prenylation, which affects several steps of signal transduction and causes various pleiotropic effects such as improving endothelial function, immune modulation, antioxidant activity, and treatment of malignancies [ 28 ]. Bacterial HMG-CoA is 10,000 times weaker than the human enzyme. Therefore, the mechanism of the hypolipidemic effect of statins, such as inhibition of HMG CoA reductase, cannot be attributed to their antibacterial activity. Previous studies have attributed the antimicrobial effect of statins to increasing the clearance of bacteria from the infected area or to increasing the apoptosis of microbial cells [ 11 , 29 ]. In addition, the hydrophobic nature of atorvastatin also explains its antibacterial effect, where it damages the bacterial membrane in a soap-like manner, causing it to disrupt. However, the exact mechanism of action needs more research [ 30 - 31 ]. 

Das *et al*. [ 32 ] investigated the antibacterial effect of atorvastatin on two periodontal pathogens, including Aa and *Porphyromonas gingivalis* (Pg), and reported that both bacteria are sensitive to this substance, which is consistent with our study. They obtained a MIC of 0.8 and 12.5μg/ml for Pg and Aa, respectively, which was much higher than the current investigation. Compared to existing literature, our conventional atorvastatin MIC (0.039µg/ml) demonstrates superior efficacy to the 12.5 µg/ml reported by their study for identical Aa strains. 

This enhancement may reflect optimized drug purity or methodological refinements. 

More notably, our nano-atorvastatin MIC (0.002µg/ ml) establishes potency against periodontal pathogens. This efficacy amplification likely stems from multifaceted mechanisms; niosomal encapsulation promotes bacterial membrane fusion, enables efflux pump evasion, and enhances biofilm penetration [ 33 - 34 ]. The hydrophobic nature of atorvastatin further synergizes with nano-delivery, creating dual-action membrane disruption, exceeding CHX efficacy (MIC: 780µg/ml) despite its clinical dominance [ 35 ].

Despite exhibiting a lower MIC than free atorvastatin, nano-atorvastatin demonstrated a slightly reduced antimicrobial effect in the well-diffusion assay. This apparent discrepancy may arise from limitations inherent in the diffusion test methodology. While MIC measures the intrinsic concentration-dependent growth inhibition in liquid broth, the well-diffusion assay critically depends on the agent's ability to diffuse effectively through the solid agar matrix. The nano formulation's physicochemical properties- including potentially larger hydrodynamic size, aggregation propensity, or interactions between the niosomal components (surfactants, cholesterol) and the agar- could significantly hinder its diffusion rate and radial spread from the well [ 20 - 21 ]. Consequently, even though nano-atorvastatin is more potent at lower concentrations (lower MIC); its restricted diffusion within the agar medium limits the observable zone of inhibition size compared to the smaller, more freely diffusible free drug molecules.

Emani *et al*. [ 36 ] showed the effect of simvastatin against Aa and Pg and suggested that Aa is more sensitive to this substance compared to Pg. In line with the current study, their investigation also found the antibacterial properties of statins. Moreover, Masadeh *et al*. [ 29 ] found that statins, including atorvastatin, simvastatin, and rosuvastatin, can induce different degrees of antibacterial activity. Additionally, Lindy *et al*. [ 37 ] assessed the effectiveness of statins (including atorvastatin and simvastatin) in patients with periodontitis and found that people treated with statins had a lesser extent (37%) of pathological periodontal pockets compared to the placebo group. Therefore, statins can reduce the activity of bacteria effective in periodontitis, including Aa, which is in line with the current study. 

On the other hand, Kadkhoda *et al*. [ 38 ] suggested that CHX 0.2% reduced the growth halo of Aa with a diameter of 17.8 mm, which was in line with the present investigation.

These findings suggest nano-atorvastatin as a promising and innovative candidate for periodontal therapy. Its ultra-low effective concentration potentially mitigates systemic side effects associated with high-dose statins. However, clinical translation requires addressing critical research gaps. The current investigation is subject to some limitations. First, the research was conducted *in vitro* and used only a specific bacterial type, which may limit the generalizability of the results. Moreover, the well-diffusion method's reliability is compromised by its dependence on variables such as the substance's solubility and diffusion rate.

Future studies must evaluate the performance of nano-atorvastatin in complex biofilm ecologies and assess its biocompatibility with oral tissues. While our *in vitro* results establish foundational efficacy, comprehensive *in vivo* validation remains essential to harness this nanotechnology breakthrough for practical periodontitis management. The results of this *in vitro* study suggest the potential efficiency of nanotechnology for managing periodontitis; however, conducting further *in vivo* studies is necessary to validate these findings. 

## Conclusion

The findings of the present *in vitro* study suggested the antibacterial properties of atorvastatin and nano-atorvastatin. However, this effect is greater with atorvastatin compared to nano-atorvastatin. Moreover, both atorvastatin and nano-atorvastatin could inhibit the growth of bacteria in a concentration of less than 1μg/ml. Therefore, these substances can be used as an additional drug in treating periodontal diseases.
